# Human dental pulp stem cells mitigate the neuropathology and cognitive decline via AKT-GSK3β-Nrf2 pathways in Alzheimer’s disease

**DOI:** 10.1038/s41368-024-00300-4

**Published:** 2024-05-13

**Authors:** Wei Xiong, Ye Liu, Heng Zhou, Junyi Li, Shuili Jing, Cailei Jiang, Mei Li, Yan He, Qingsong Ye

**Affiliations:** 1 Center of Regenerative Medicine, Department of Stomatology, Renmin Hospital of Wuhan University, Wuhan, Hubei, China; 2 Institute of Regenerative and Translational Medicine, Tianyou Hospital, Wuhan University of Science and Technology, Wuhan, China; 3 Department of Oral Science, Faculty of Dentistry, University of Otago, Dunedin, New Zealand; 4 Department of Oral and Maxillofacial Surgery, Massachusetts General Hospital, Harvard Medical School, Boston, MA USA; 5 Department of Stomatology, Linhai Second People’s Hospital, Linhai, Zhejiang, China

**Keywords:** Mesenchymal stem cells, Ageing

## Abstract

Oxidative stress is increasingly recognized as a major contributor to the pathophysiology of Alzheimer’s disease (AD), particularly in the early stages of the disease. The multiplicity advantages of stem cell transplantation make it fascinating therapeutic strategy for many neurodegenerative diseases. We herein demonstrated that human dental pulp stem cells (hDPSCs) mediated oxidative stress improvement and neuroreparative effects in in vitro AD models, playing critical roles in regulating the polarization of hyperreactive microglia cells and the recovery of damaged neurons. Importantly, these therapeutic effects were reflected in 10-month-old 3xTg-AD mice after a single transplantation of hDPSCs, with the treated mice showing significant improvement in cognitive function and neuropathological features. Mechanistically, antioxidant and neuroprotective effects, as well as cognitive enhancements elicited by hDPSCs, were at least partially mediated by Nrf2 nuclear accumulation and downstream antioxidant enzymes expression through the activation of the AKT-GSK3β-Nrf2 signaling pathway. In conclusion, our findings corroborated the neuroprotective capacity of hDPSCs to reshape the neuropathological microenvironment in both in vitro and in vivo AD models, which may be a tremendous potential therapeutic candidate for Alzheimer’s disease.

## Introduction

Dementia and related neurodegenerative diseases have become important determinants concerning health among geriatric populations around the world. As the most common cause of dementia, Alzheimer’s disease (AD) is a highly disabling disorder characterized by progressive neurodegeneration and irreversible cognitive impairment, making up approximately 60% to 80% of global dementia cases.^1^ However, despite high prevalence and extensive research efforts, there are currently no curative treatments that can prevent and/or delay disease progression, underscoring the urgent need for novel therapeutic strategies.

Age is a significant risk factor for developing AD. The hallmark pathological features observed in AD brains include senile plaques, neurofibrillary tangles, oxidative stress, and neuroinflammation, which are exacerbated in an age-dependent manner.^2^ A growing amount of evidence suggests that oxidative stress induced by imbalances in redox status appears to be a critical driving force contributing to the progression of AD.^2–4^ Oxidative stress, characterized by excessive production of harmful reactive oxygen species (ROS), causes mitochondrial damage and dysfunction, which accelerates weaker synaptic connections and neuronal necrosis.^5,6^ Nuclear factor erythroid-derived 2-related factor 2 (Nrf2) is the principal transcription factor involved in the regulation of redox homeostasis, stabilizes and translocations to the nucleus under stressful conditions, binds to antioxidant response elements (AREs), and regulates the expression of various detoxification and antioxidant genes, including heme oxygenase-1 (HO-1).^7,8^ The available evidence suggests that in AD patients or animal models of the hippocampus, Nrf2 activity is significantly reduced and is predominantly located in the cytoplasm rather than being evenly distributed between the nucleus and cytoplasm as in healthy individuals.^9,10^ Given these etiological features, developing new interventions to reduce cellular oxidative stress in the AD brain should be of concern, albeit challenging.

Recently, stem cell-based regenerative therapies have emerged as one of the most promising and exciting techniques for AD treatment. In this context, mesenchymal stem cells (MSCs) with neurotrophic factor release and potent immunomodulatory properties have been highlighted as a new approach to AD cell therapy.^11^ Accumulating research investigations have indicated that transplantation of MSCs, such as adipose mesenchymal stem cells (ADMSCs), human umbilical cord-derived mesenchymal stem cells (huc-MSCs) or bone marrow stem cells (BMSCs), into the brain significantly improved cognitive dysfunction and pathological symptoms in AD animal models.^12,13^ However, several factors have led to major limitations in their clinical application, including invasive routine collection procedures, low yields, and ethical concerns.

Here, our group used a more readily available population of MSCs, i.e., human dental pulp stem cells (hDPSCs) originated from the cranial neural crest, which are usually derived from extracted teeth for orthodontic purposes or impacted third molars. Based on established in vitro AD cell models and in vivo therapeutic application, we were designed to explore the potential therapeutic effect of hDPSCs on the oxidative stress and damaged neurons in the AD microenvironment. Specifically, we demonstrated that hDPSCs significantly improved cognitive decline and intracranial microenvironment in AD mice and that the beneficial effects were largely dependent on AKT/GSK3β-mediated Nrf2 activation and nuclear accumulation.

## Results

### hDPSCs mitigated LPS-triggered inflammation in vitro

Human dental pulp stem cells (hDPSCs) isolated from the teeth of healthy volunteers are characterized by multi-differentiation potential and MSC-like surface markers. HDPSCs penetrated out from dental pulp tissue fragments and proliferated to form dense colonies that present typical fibroblastic morphology (Figure S1a, P0-P3). The multipotency of hDPSCs were identified by osteogenic, chondrogenic, and adipogenic differentiation. After staining with Alizarin Red, Alcian Blue, and Oil Red O, the results showed that the presentation of calcium phosphate, proteoglycans, and lipid droplets under the corresponding induced cues (Figure S1b). Flow cytometry results performed to evaluate the MSC-like properties of hDPSCs also showed that hDPSCs expressed CD44, CD73, and CD90 positively and CD34, CD45, and HLA-DR negatively (Figure S1c).

**Fig. 1:**
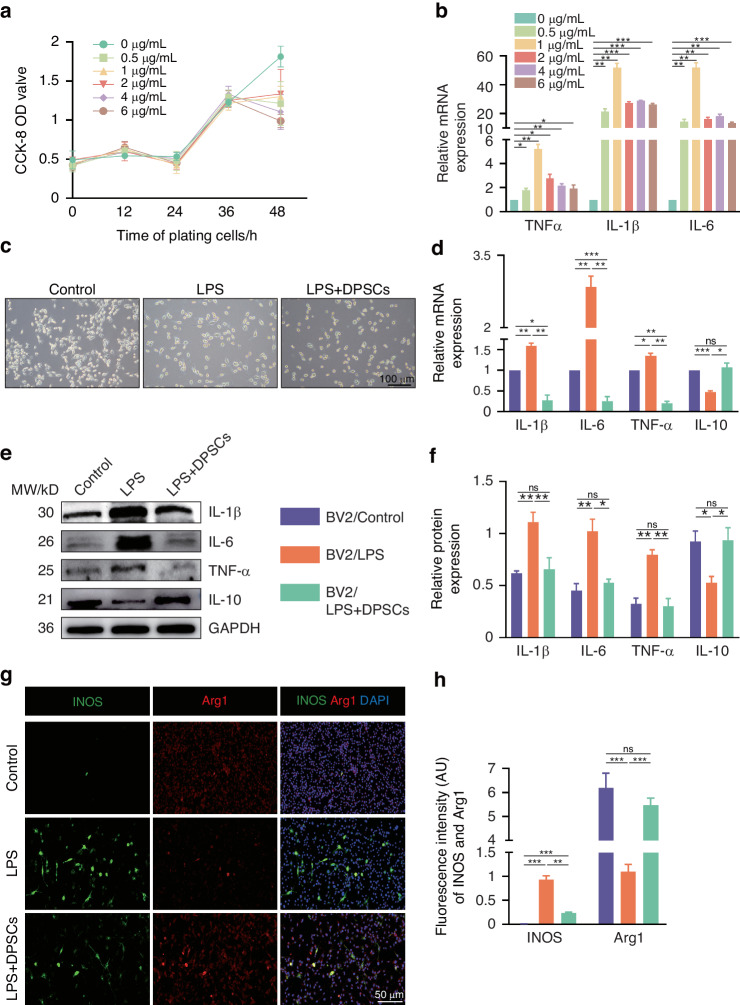
hDPSCs mitigated LPS-triggered inflammation in vitro. **a** Primary BV2 cells were induced at different time by increasing doses of LPS (0–6 μg/ml), then cell viability was measured by CCK-8 assay. **b** The mRNA expression of pro-inflammatory cytokine (TNF-α, IL-1β, and IL-6) in LPS-induced BV2 cells for 24 h. **c** The representative morphological images of BV2 cells in Control group, LPS-induced group and hDPSCs co-culture group were observed under light microscope. Scale =100 μm. **d** The mRNA expression of TNF-α, IL-1β, IL-6, and IL-10 in BV2 cells of three group. **e**, **f** Representative images and quantification of western blotting showed the expression of inflammatory-related factors (TNF-α, IL-1β, IL-6, and IL-10) in different treated BV2 cells. **g**, **h** Representative immunostaining images and quantification of markers for activated microglia (INOS, green and Arg1, red). Scale = 50 μm (*n* = 3 per group; Values represented mean ± SD; ns indicates no significant, *P < 0.05, **P < 0.01, ***P < 0.001)

Prolonged periods of uncontrolled intracerebral homeostatic imbalances lead to abnormal activation of microglia and chronic neuroinflammation, which can further exacerbate neurodegeneration and cognitive impairment.^1,14^ To investigate the effects of hDPSCs on microglia abnormal activation pathology, we induced BV2 cells with bacterial lipopolysaccharide (LPS) stimulation, which is considered the gold standard for microglia activation.^15^ BV2 cells were challenged with different conditions of LPS to determine the best induced effects. As previously described,^16,17^ BV2 cells were incubated with LPS at a final concentration of 1ug/ml for 24 h, resulting in decreased cell viability and significantly increased expression of pro-inflammatory factors (Fig. 1a, b). Light microscope results of LPS-induced BV2 cells in vitro showed that the cell morphology of the LPS-induced BV2 cells exhibited a branching morphology and larger volumes compared with normal group; Co-cultured with hDPSCs reversed the stimulatory reactivity (Fig. 1c). In AD, microglia, which originally play a neuroprotective role by degrading Aβ plaques through phagocytosis, are abnormally activated into a pro-inflammatory state and release various neurotoxic molecules.^15,18^ We next investigated whether hDPSCs influenced microglia reactivity and homeostasis by measuring levels of inflammatory factors and phenotypic changes associated with morphological activation. We found a strong reduction in mRNA levels of pro-inflammatory cytokines (IL-1β, IL-6, and TNF-α) in microglia after co-culture with hDPSCs, whereas the mRNA expression of anti-inflammatory cytokines (IL-10) was enhanced (Fig. 1d). Similar to the positive efficacy of hDPSCs regulating inflammatory cytokines at the transcriptional level, western blot also confirmed that hDPSCs significantly reduced the protein expression of LPS-induced pro-inflammatory cytokines in microglia (Fig. 1e, f). Intriguingly, the mRNA expression of pro-inflammatory cytokines in the hDPSCs-treatment group decreased even more than that in the WT group, which may be closely related to the strong regulation of transcription by stem cells. Considering that LPS is thought to be a representative inducer of microglial polarization,^19^ in this co-culture model, we also assessed whether hDPSCs affected microglial polarization in inflammatory injury conditions. In line with this, the immunostaining for the M1-phenotype marker INOS was decreased and the M2-phenotype marker Arg1 was significantly increased in the hDPSCs-treatment group than that in LPS group (Fig. 1g, h), suggesting that hDPSCs reduced the number of hyperinflammation-induced activated microglia, promoting M1-reactive microglia tendencies toward the M2-protective phenotype. In the control group of BV2 cells, there was little evidence of INOS-positive cells. These results demonstrated that when faced with a hyperinflammatory challenge, hDPSCs could reverse microglial hyperreactive states and mitigate inflammation levels.

### hDPSCs ameliorated LPS-induced oxidative stress and apoptosis in BV2 cells by activating Nrf2 via the AKT/GSK3β pathway

**Fig. 2:**
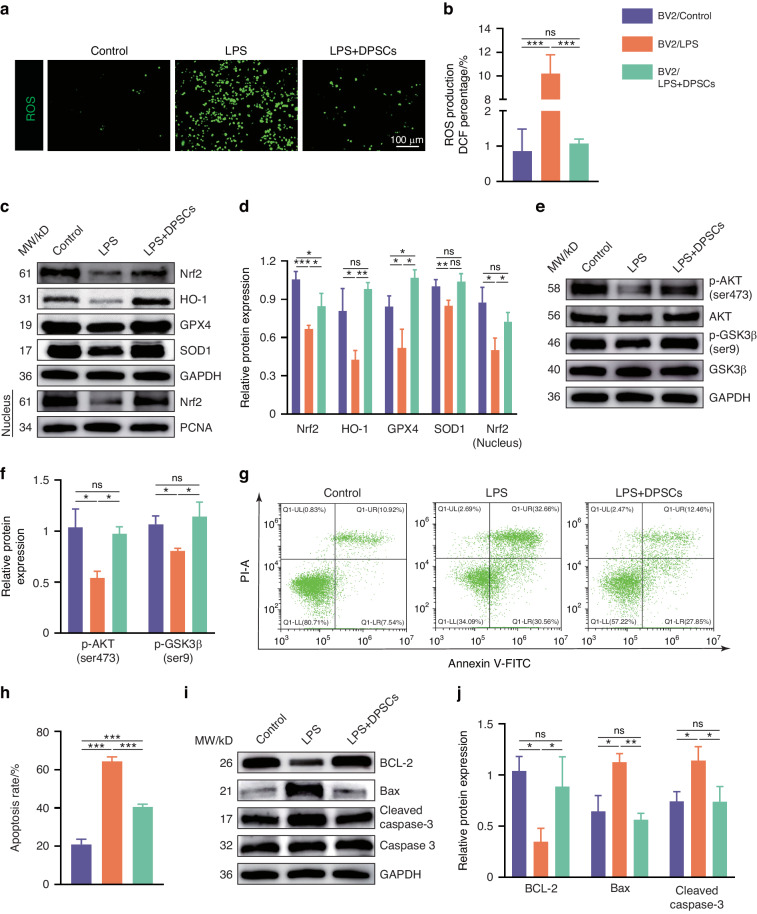
hDPSCs ameliorated LPS-induced oxidative stress and apoptosis in BV2 cells by activating Nrf2 via the AKT/GSK3β pathway. **a**, **b** The reactive oxygen species (ROS, green) level in BV2 cells detected by DCFH-DA staining and statistically analyzed. Scale = 100 μm. **c**, **d** Representative images and quantification of western blotting showed the expression of total Nrf2, HO-1, GPX4, SOD1, and nuclear Nrf2 in different treated BV2 cells. **e** Representative western blotting results showed the expression of p-AKT (ser473) and p-GSK3β (ser9) of BV2 cells in Con, LPS, and LPS+hDPSCs groups. **f** The quantification of p‐AKT (ser473) and p‐GSK3β (ser9), respectively, compared with total‐AKT and total‐GSK3β. **g**, **h** The apoptosis of BV2 cells in each group was determined by flow cytometry. **i**, **j** Representative images and quantification of western blotting showed the expression of BCL-2, Bax, and cleaved caspase 3 in different treated BV2 cells (*n* = 3 per group; Values represented mean ± SD; ns indicates no significant, *P < 0.05, **P < 0.01, ***P < 0.001)

It has been shown that microglia or macrophages exposed to inflammatory stimuli undergo metabolic reprogramming, facilitating cytokine and ROS production.^20^ Given the downregulation of the pro-inflammatory genes, we suspected that hDPSCs modulated the microglial phenotypic transition by blocking the ROS-triggered inflammatory signaling pathway. Differences in microglial reactivity were further supported by changes in ROS after LPS or hDPSCs treatment, noted by an increased number of DCF-positive cells in the LPS-induced BV2 cells, and hDPSCs predominantly inhibited ROS production (Fig. 2a). The DCF fluorescence quantitative analysis also confirmed the result (Fig. 2b).

Considering that we observed that hDPSCs significantly reduced LPS-induced neuroinflammation and ROS production in BV2 cells, we next hypothesized that hDPSCs might mediate the expression of relevant antioxidant enzymes, which are known as pivotal molecules in regulating oxidative stress and inflammation.^21^ RT-PCR assays manifested that consistent with foregoing experimental results, hDPSCs inhibited the decline in mRNA expression of the antioxidant factors Nrf2, HO-1, SOD1, and GPX4 (Figure S2a). Among them, Nrf2 acts as a major transcriptional regulator and translocates from the cytoplasmic to the nucleus to increase the activity of antioxidant response kinases, including H0-1, SOD, and GPX4. Meanwhile, the functional activation of Nrf2 is also controlled by upstream regulators phosphorylated protein kinase B (AKT) and phosphorylated glycogen synthase kinase 3β (GSK3β).^8,22^ Therefore, we further verified the protein expression of these genes. Western blotting revealed that hDPSCs significantly suppressed LPS-induced downregulation of Nrf2, HO-1, GPX4, and SOD1 by promoting the upregulation of p-AKT (ser473)/p-GSK3β (ser9) and the nuclear translocation of its downstream Nrf2 (Fig. 2c–f). High doses of LPS are well known to cause the production of inflammatory mediators and damaging molecules, but they also lead to toxic injury to the cell itself.^19,23^ Based on the AnnexinV-PI apoptosis experiment, we also verified that hDPSCs ameliorated LPS-induced BV2 cells damage because we observed a decrease in the percentage of apoptosis in the hDPSCs treatment group, although not as much as in the normal group (Fig. 2g, h). In line with this, further analyses found that in LPS-induced BV2 cells, co-culture with hDPSCs significantly increased the expression of the anti-apoptotic protein BCL2 and decreased the expression of apoptotic proteins cleaved caspase 3 and Bax (Fig. 2i, j).

To further confirm whether the AKT/GSK3β signaling pathway is critical for hDPSCs-induced nuclear accumulation of Nrf2, BV2 cells were preincubated with an AKT pathway inhibitor for 2 h, followed by hDPSCs treatment,^24^ and then the associated protein expression was detected. Western blot results showed that LY294002 significantly diminished hDPSCs-induced phosphorylation of p-AKT (ser473) and p-GSK3β (ser9) compared with hDPSCs-treated groups, which caused decreased protein expression of Nrf2 and its downstream gene HO-1 and reduced nuclear accumulation of Nrf2 (Figure S3a–d). Downregulation of LY294002-induced expression of antioxidant proteins also resisted the elimination of ROS by hDPSCs in LPS-induced BV2 cells (Figure S3e, f). Generally, these results demonstrated that hDPSCs can mitigate LPS-induced microglial damage by activating the AKT/GSK3β pathway, which enables the accumulation of Nrf2 in the nucleus to contain oxidative stress and neuroinflammation.

### hDPSCs alleviated oxidative stress and mitochondrial damage in GLU-treated HT22 cells

**Fig. 3:**
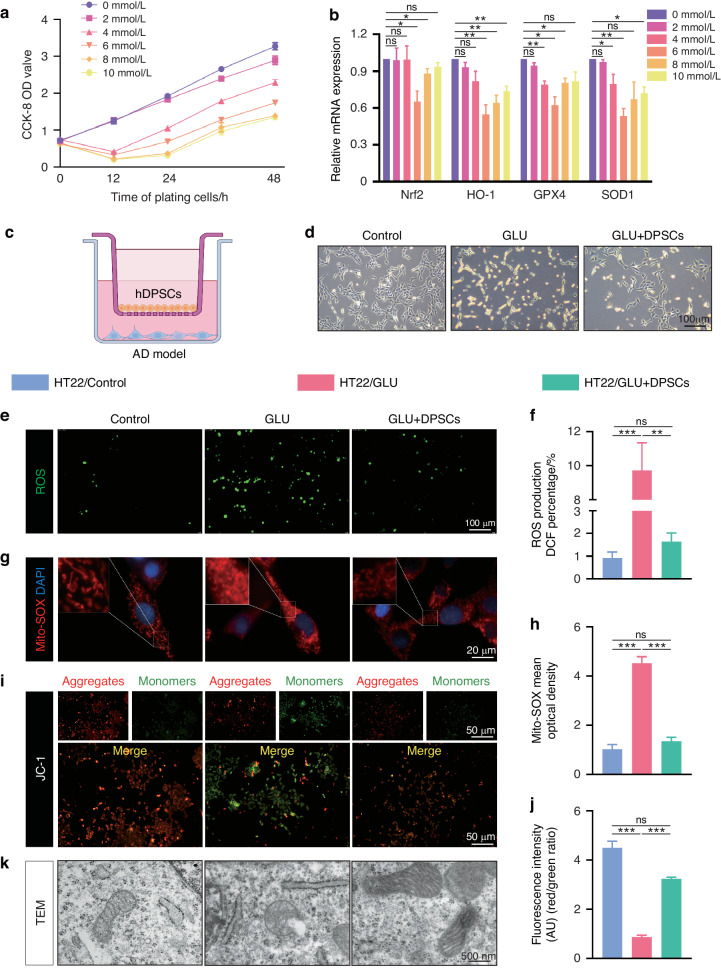
hDPSCs alleviated oxidative stress and mitochondrial damage in GLU-treated HT22 cells. **a** Primary HT22 cells were induced at different time by increasing doses of GLU (0–10 μg/mL), then cell viability was measured by CCK-8 assay. **b** The mRNA expression of antioxidant cytokine (Nrf2, HO-1, GPX4, and SOD1) in GLU-induced HT22 cells for 24 h. **c** A transwell non-contact co-culture assay system was structured into the AD model. **d** The representative morphological images of HT22 cells in Control group, GLU-induced group, and hDPSCs co-culture group were observed under light microscope. Scale = 100 μm. **e**, **f** The reactive oxygen species (ROS, green) level in HT22 cells detected by DCFH-DA staining and statistically analyzed. Scale = 100 μm. **g**, **h** The mitochondrial ROS level in HT22 cells determined by MitoSOX red staining. Scale = 100 μm. **i**, **j** Mitochondrial membrane potential of the HT22 cells were detected by the JC-1 staining and the quantification of red/green fluorescence intensity. Scale bar = 50 μm. **k** Morphometric ultrastructural analyses by TEM showed the intracellular mitochondrial structure of HT22 in the three groups. Scale bar = 500 nm (*n* = 3 per group; values represented mean ± SD; ns indicates no significant, *P < 0.05, **P < 0.01, ***P < 0.001)

Pro-inflammatory M1 microglia not only produce pro-inflammatory factors and ROS but also enhance the excitotoxicity of glutamate, thus damaging neurons and the brain microenvironment.^25^ During the progression of AD, excessive extracellular glutamate concentrations stimulate glutamate receptors, which not only leads to intracellular calcium overload but also contributes to the production of ROS, resulting in cellular excitotoxicity and neuronal death.^26,27^ Therefore, based on the demonstrated ability of hDPSCs to shift microglia toward an anti-inflammatory phenotype and provide protection, we further examined whether hDPSCs have a neuroprotective effect in a glutamate-induced excitotoxicity in vitro AD model. As shown in Fig. 3a, b, CCK-8 and RT-PCR experiments were first performed to determine the optimal concentration of 6 mM/L glutamate to induce HT22 cells for 12 h. Using the co-culture models with hDPSCs and glutamate-induced HT22 (GLU group) (Fig. 3c), we observed the cell morphology by light microscopy and found that most of the neurons that lost their original cell morphology after being stimulated by glutamate gradually recovered normal cell morphology similar to that of the control group after co-culture with hDPSCs (Fig. 3d). Similar to the results of LPS-induced BV2 cells, HT22 cells exposed to glutamate clearly increased DCF-positive cells, while co-culture with hDPSCs obviously inhibited intracellular ROS production (Fig. 3e, f).

A closely association between physiological aging and declining mitochondrial function has long been noted.^28^ The genetic polymorphisms present in the etiology of a vast majority of patients with late-onset AD are also associated with age-dependent oxidative stress and mitochondrial abnormalities.^29^ Glutamate excitotoxicity induces an intracellular Ca^2+^ superload that impairs mitochondrial antioxidant activity and increases mitochondrial ROS production, ultimately leading to apoptosis.^30,31^ Next, we also tested whether hDPSCs also protect mitochondria. As evidenced by Mito-SOX red staining results, high concentrations of glutamate lead to ROS accumulation in the mitochondria of neuronal cells, and hDPSC significantly alleviated this characterization (Fig. 3g, h). Synchronously, to further evaluate the effect of hDPSCs on oxidative stress-induced mitochondrial damage, JC-1 staining, and transmission electron microscopy (TEM) were used to detect mitochondrial membrane potential (MMP) and structural integrity, respectively. As depicted in Fig. 3i, j, compared with GLU-induced HT22 cells, the depolarization of mitochondrial membrane potential was distinctly attenuated after hDPSCs treatment. Morphometric analyses with TEM indicated that the intracellular mitochondria of GLU-damaged HT22 cells presented swollen and vacuolated, whereas treatment with hDPSCs restored the mitochondria to a long shape resembling that of the WT group, and the mitochondrial cristae were also arranged neatly (Figs. 3k, S4). Taken together, these data illustrated that hDPSCs alleviated oxidative stress and mitochondrial damage in GLU-treated HT22 cells.

### hDPSCs attenuated apoptosis in GLU-induced HT22 cells by activating Nrf2 via the AKT/GSK3β pathway

**Fig. 4:**
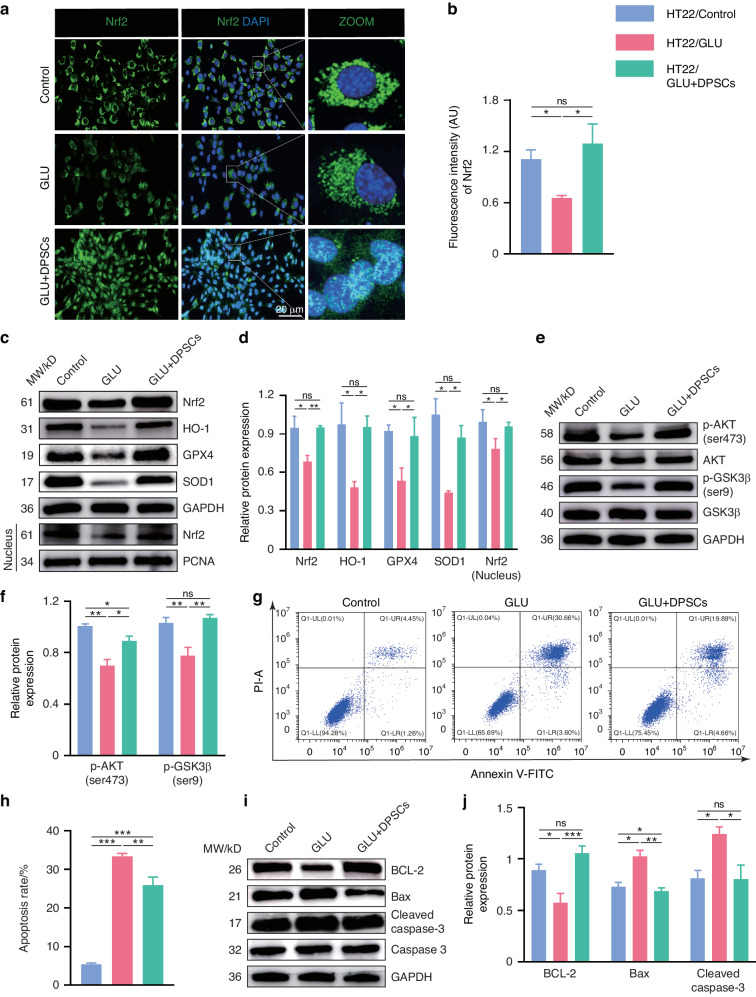
hDPSCs attenuated apoptosis in GLU-induced HT22 cells by activating Nrf2 via the AKT/GSK3β pathway. **a**, **b** Representative immunofluorescent staining images and quantification of Nrf2 in three different groups. Scale = 20 μm. **c**, **d** Representative images and quantification of western blotting showing the expression of total Nrf2, HO-1, GPX4, SOD1, and nuclear Nrf2 in different treated HT22 cells. **e** Representative western blotting results showed the expression of p-AKT (ser473) and p-GSK3β (ser9) of HT22 cells in the three groups. **f** The quantification of p‐AKT (ser473) and p‐GSK3β (ser9), respectively, compared with total‐AKT and total‐GSK3β. **g**, **h** The apoptosis of HT2 cells in each group was determined by flow cytometry. **i**, **j** Representative images and quantification of western blotting showing the expression of BCL-2, Bax, and cleaved caspase 3 in different treated HT22 cells. (*n* = 3 per group; Values represented mean ± SD; ns indicates no significant, *P < 0.05, **P < 0.01, ***P < 0.001)

Next, considering the antioxidant activity of Nrf2 in BV2 cells, we conducted a more in-depth investigation of whether the antioxidant effect of hDPSCs is mediated by Nrf2 in GLU-induced HT22 cells. After co-culture of hDPSCs with GLU-treated HT22 cells, immunofluorescence staining and quantitative analysis showed that the overall Nrf2 expression level of neuronal cells treated with hDPSCs was higher than that of GLU group, and the accumulation/translocation of Nrf2 within the nucleus was significantly enhanced in hDPSCs-treated group (Fig. 4a, b). Similar results were verified by RT-PCR and western blot analysis, which showed that hDPSCs not only up-regulated the mRNA and total protein expression of Nrf2 and downstream antioxidant enzymes including HO-1, SOD1, and GPX4, but also increased the nuclear protein expression level of Nrf2 (Figs. 4c, d and S2b).

Furthermore, to determine whether hDPSCs still exert neuroprotection by activating Nrf2 via the AKT/GSK3β pathway, the expression of related gene and cell apoptosis were measured. As anticipated, compared with the GLU-treated group, hDPSCs induced the activation of p-AKT (ser473), resulting in the inhibition (increased phosphorylation) of GSK3β, accompanied by the enhanced accumulation and stabilization of Nrf2 in the nucleus (Fig. 4e, f). Then, after treating GLU-induced HT22 cells with hDPSCs, the apoptosis rate level was determined by flow cytometry, while the expression of apoptosis-related proteins was examined by western blotting. We found that the apoptosis rate of HT22 cells induced by GLU was strongly reduced after co-culture with hDPSCs (Fig. 4g, h). Not only that, but the western blot analysis also showed that hDPSCs significantly up-regulated the expression of the anti-apoptotic protein BCL2 and decreased the expression of the pro-apoptotic protein Bax and cleaved caspase 3 (Fig. 4i, j). Preincubation of LY294002 similarly confirmed that, compared to the hDPSCs-treated group, the increased protein expression of p-AKT (ser473), p-GSK3β (ser9), nuclear Nrf2, and HO-1 were reversed by LY294002 (Figure S5a–d). This inhibition of the antioxidant effect of hDPSCs ultimately led to an over-release of ROS (Figure S5e, f). In summary, the cumulative data revealed that at least to some extent, hDPSCs exert a neuroprotective effect by promoting nuclear accumulation and stabilization of Nrf2 via the p-AKT (ser473)/p-GSK3β (ser9) pathway in vitro AD cell model.

### Administration of hDPSCs enhanced spatial learning and memory ability in 3xTg-AD mice

**Fig. 5:**
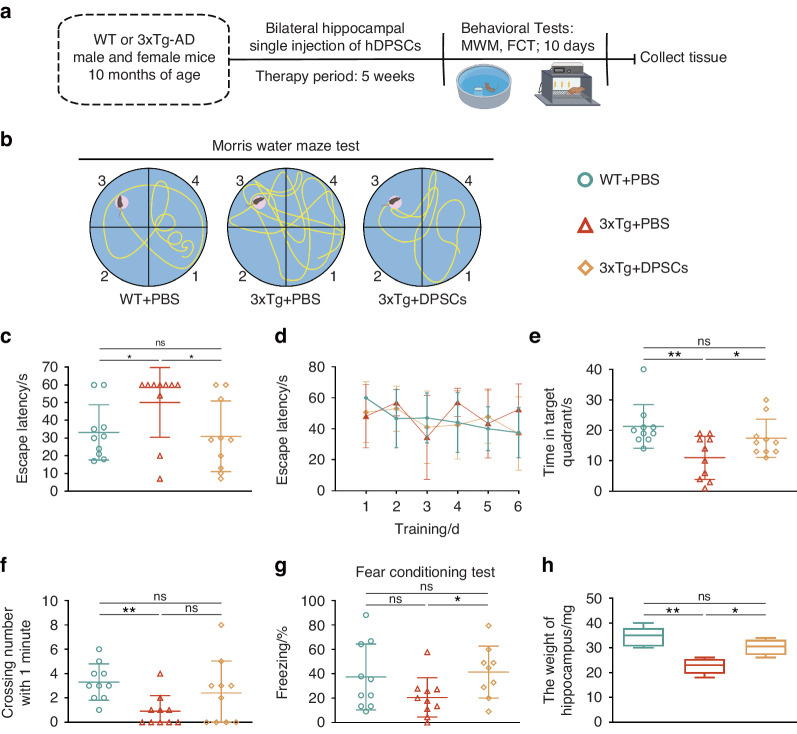
Administration of hDPSCs enhanced spatial learning and memory ability in 3xTg-AD mice. **a** Experimental design is illustrated schematically. **b**–**f** Results from WT-PBS, AD-PBS, and AD-DPSCs groups mice that were cognitively tested by MWM. The typical escape paths (**b**), the escape latency(s) (**c**), the time mice swam in the target quadrant (**e**), and the average number of crossing previous platform location in hidden‐platform test on the seventh day. **d** Training curves showed the average escape latency(s) to the hidden platform during the first 6 days acquisition training. **g** Comparison and quantification of the freezing rate (%) in the three groups of mice participating in the fear conditioning test. **h** Quantitative comparison of hippocampal compartments of isolated mice after behavioral testing (*n* = 3–10 per group; Values represented mean ± SD; ns indicates no significant, *P < 0.05, **P < 0.01)

It is well known that in the onset and development of AD, whether it is oxidative stress, neuroinflammation or nerve cell dysfunction, it will eventually cause cognitive decline in AD patients. Therefore, to further explore whether hDPSCs can enhance the memory and cognitive functions of AD mice, behavioral tests including the Morris water maze (MWM) and fear conditioning test (FCT) were performed. As shown in the experimental diagram, animals were first tested in the MWM task 5 weeks after the single transplantation of hDPSCs into the bilateral hippocampus of 10-month-old 3xTg-AD mice (Fig. 5a). During the preceding 6 days of training, the AD-phosphate buffered saline (AD-PBS) mice spent more time reaching the escape platform compared with the WT and hDPSCs-treated mice (Fig. 5d). Consistent with this, the WT and hDPSCs-treated mice showed better performance in the escape latency time than that of AD-PBS mice during the test period (Fig. 5c), and the typical escape route showed a clear orientation rather than the erratic swimming path of AD-PBS mice (Fig. 5b). Moreover, after removal of the hidden platform, hDPSCs-treated mice spent longer exploration time in the target quadrant and crossed the original platform location more times within 60 s compared to the AD-PBS mice (Fig. 5e, f). In 2 days of the fear conditioning test, the freezing rate of the hDPSCs-treated mice was only slightly, albeit statistically significant, higher than that of AD-PBS mice (Fig. 5g). Astonishingly, when mouse hippocampal tissue was collected after behavioral testing, we found that the hippocampal mass of AD + PBS mice was significantly less than that of WT mice, and this difference was reversed in the hDPSCs-treated mice (Fig. 5h). These results indicated that the transplantation of hDPSCs had improved impaired cognitive function in 3xTg-AD mice.

### hDPSCs reduced neuropathology in the hippocampus of 3xTg-AD mice

**Fig. 6:**
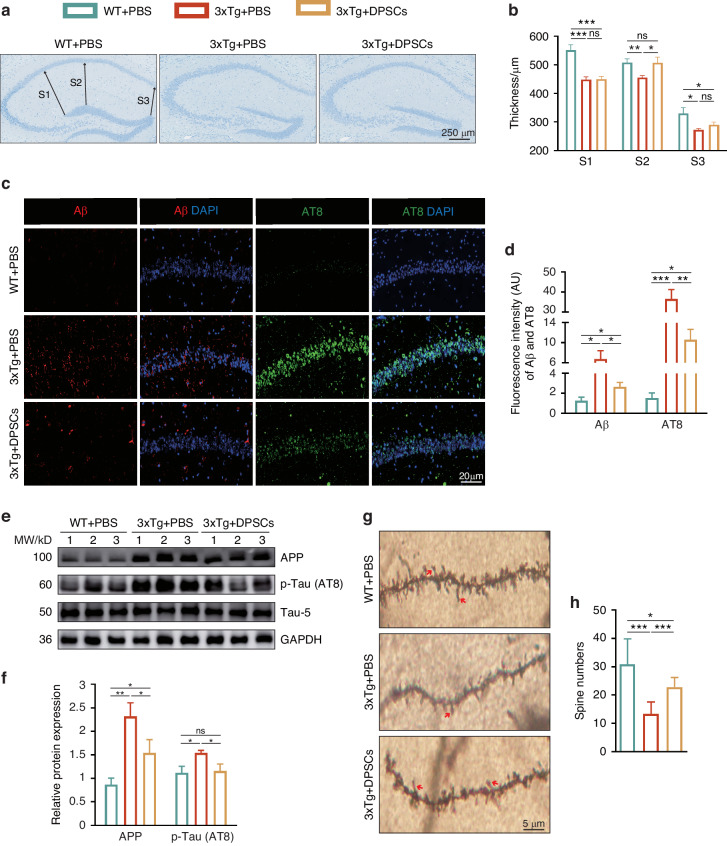
hDPSCs reduced neuropathology in the hippocampus of 3xTg-AD mice. **a** The NISSL-stained brain slice images showed arrows at S1, S2, and S3, indicating the thickness between the CA1 and DG subregions of the hippocampus. Scale = 250 μm. **b** Quantification of thickness for the 3 zones selected. **c** Aβ and AT8 immunofluorescent staining showed the extent of Aβ aggregation and Tau phosphorylation in the hippocampus between three groups mice. Scale = 20 μm. **d** Quantification of the Aβ- and AT8-marked areas in the hippocampus, respectively. **e**, **f** Representative images and quantification of western blotting showing the expression of APP and p-Tau (AT8) in the hippocampus between three groups mice. **g**, **h** Representative Golgi staining images and quantification of spine density (red arrows) in the hippocampus from mice. Scale = 5 μm. (*n* = 3 per group; Values represented mean ± SD; ns indicates no significant, *P < 0.05, **P < 0.01, ***P < 0.001)

To determine whether improvement in cognitive function in AD mice was accompanied by changes in representative pathological features in the brain, we assessed them focusing on the mouse hippocampus. Compared to healthy individuals, patients with AD have significantly reduced brain volume, which is strongly linked to atrophy caused by synaptic degeneration and neuronal death, particularly in the vulnerable hippocampus.^32,33^ We systematically compared and measured the distance between the CA1 and DG regions in the hippocampus from different groups of mice at 3 different levels (S1, S2, and S3) through the NISSL-stained brain tissue slices as indicators of hippocampal atrophy.^34^ As shown in Fig. 6a, b, hippocampal atrophy was distinctly macroscopic in AD-PBS mice compared to WT, as confirmed by a significant reduction in S1-S3. Treatment of AD mice with hDPSCs resulted in a significant higher S2 thickness, whereas little change was detected in the other two markers. The detection of apoptosis-related genes in the hippocampus also revealed that the expression of anti-apoptotic protein (BCL2) was significantly increased, and the expression of pro-apoptotic proteins Bax and cleaved caspase 3 was decreased after hDPSCs treatment (Figure S6a, b).

Since the most prominent molecular pathology of AD is the neurotoxicity of aggregate Aβ and hyperphosphorylated tau protein, which jointly damage neurons and ultimately lead to cognitive decline, we performed immunofluorescence staining to assess whether hDPSCs had modified their progression.^1^ The results revealed that the pathological Aβ aggregation and the expression of phosphorylated Tau protein (AT8) in the hippocampus of hDPSCs-treated AD mice were much lesser than those in AD-PBS mice (Fig. 6c, d). Consistent with this, Western blotting showed that hDPSCs also reduced the protein expression of APP and p-Tau (AT8) in the hippocampus of 3xTg-AD mice (Fig. 6e, f).

We next performed Golgi staining of hippocampus to determine whether hDPSCs could affect the dendritic spines, which are the primary locus of most glutamatergic excitatory synaptic interactions within neuronal circuits.^35^ Consistent with the aforementioned pathologic findings, we found a significant decrease in the spine density of secondary dendritic branches of apical dendrites in CA1 regions of AD-PBS mice compared to WT mice, whereas hDPSCs treatment prevented spine loss pathology (Fig. 6g, h). Unexpectedly, we also observed that both the complexity and integrity of neurite arborization in the CA1 and DG regions of the hDPSCs-treated mice were visibly higher than those in the AD-PBS mice (Figure S7). These data together confirmed that single administrations of hDPSCs were indeed effective in improving hippocampal neuropathology in 3xTg-AD mice.

### Neuroprotective efficacy of hDPSCs in 3xTg-AD mice was associated with enhancing Nrf2 nuclear accumulation via AKT/GSK3β pathway

**Fig. 7:**
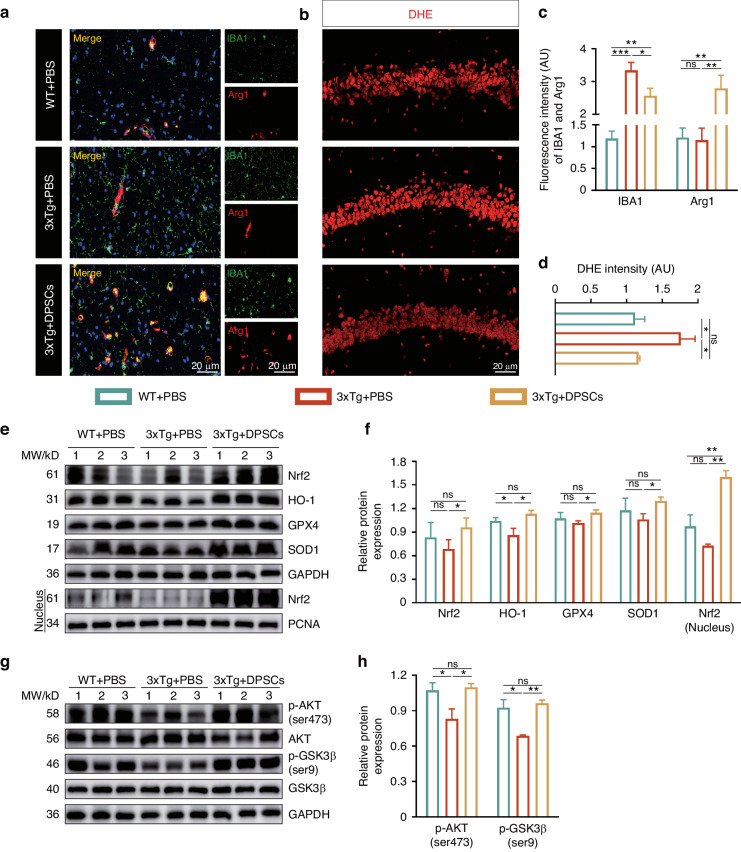
Neuroprotective efficacy of hDPSCs in 3xTg-AD mice was associated with enhancing Nrf2 nuclear accumulation via AKT/GSK3β pathway. **a**, **c** Immunofluorescence staining images and quantification of microglia (IBA1, Arg1) and IBA1-Arg1 co-localization (Merge, M2 microglia) in the hippocampus between three groups mice. Scale = 20 μm. **b**, **d** The ROS level in the hippocampus determined by DHE staining. Scale = 20 μm. **e**, **f** Representative images and quantification of western blotting showing the expression of total Nrf2, HO-1, GPX4, SOD1, and nuclear Nrf2 in different treated AD mice. **g** Representative western blotting results showed the expression of p-AKT (ser473) and p-GSK3β (ser9) of three groups mice in the three groups. **h** The quantification of p‐AKT (ser473) and p‐GSK3β (ser9), respectively, compared with total‐AKT and total‐GSK3β. (*n* = 3 per group; values represented mean ± SD; ns indicates no significant, *P < 0.05, **P < 0.01, ***P < 0.001)

After characterizing the efficacy of hDPSCs in AD models in vivo, we focused on exploring the mechanism by which hDPSCs-modulated recovery of cognitive function and neuropathological damage. Given that initial exploration of therapeutic models in vitro has shown that administration of hDPSCs improved microglial polarization and neuronal oxidative stress imbalance, which are closely linked to brain microenvironmental homeostasis such as Aβ clearance and neuronal normal communication, we expanded on these findings. By immunofluorescence, compared with AD-PBS mice, the hyperreactive microglia (IBA1^+^, INOS^+^) in the hippocampus were significantly decreased and M2 microglia marker expression (IBA1^+^, Arg1^+^) increased after 5 weeks of hDPSCs transplantation, suggesting that the administration of hDPSCs promoted the M2 polarization of microglia, which contributes to the clearance of Aβ and neuroinflammation in the AD mice brain (Figs. 7a, c, and S8a, b). DHE staining also indicated that hDPSCs remarkably diminished the ROS generation in the hippocampus of AD mice, basically consistent with normal ROS levels in WT mice (Fig. 7b, d). To determine whether engraftment of hDPSCs influences the activation of Nrf2, levels of Nrf2 and downstream targets were analyzed in the hippocampus of 3xTg-AD mice. Immunofluorescence staining of hippocampal slices revealed a significant increase in the number of Nrf2-positive cells in the hDPSCs-treated AD mice, even higher than in wild-type mice, with a marked nuclear translocation (Figure S8c, d). Western blot and quantitative analysis of hippocampal tissue also showed that the protein expression of Nrf2, HO-1, and other antioxidant-related genes in hDPSCs-treated AD mice was increased compared with that in AD-PBS mice (Fig. 7e, f).

To further determine whether the mechanism of the suppressed oxidative stress and the activation of Nrf2 in the hippocampus is consistent with in vitro models, we examined the protein expression of the AKT/GSK3β pathway. The results revealed that treatments with hDPSCs overcame these negative effects by promoting nuclear accumulation of Nrf2 via stimulating the positive effects of phosphorylation of AKT and GSK3β (Fig. 7g–h). In conclusion, we demonstrated that transplantation of hDPSCs improved the oxidative stress microenvironment in the hippocampus of 3xTg-AD mice by activating the AKT/GSK3β/Nrf2 pathway, providing excellent neuroprotective efficacy.

## Discussion

**Fig. 8:**
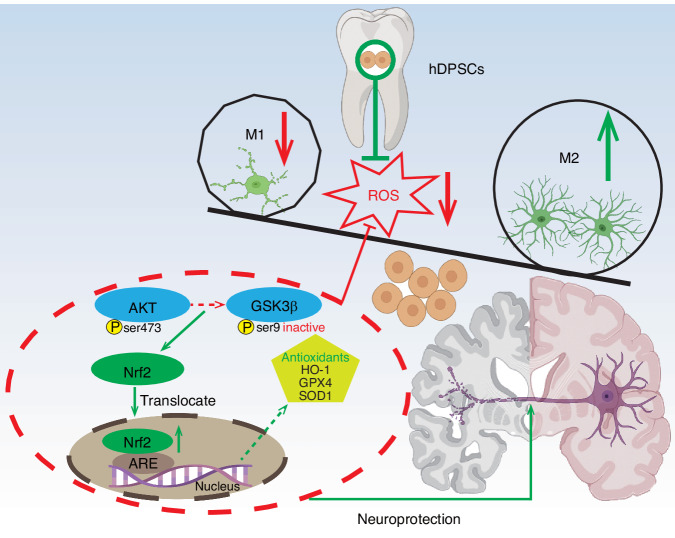
Schematic diagram summarizing the proposed mechanisms for the neuroprotective effects of hDPSCs in the Alzheimer’s disease (AD) brain. The mechanisms of application of hDPSCs in AD models involve the effects of M1/M2 polarization in microglia, promotion of antioxidant enzymes expression, inhibition of ROS production, and the role of p-AKT (ser473)/p-GSK3β (ser9)-mediated Nrf2 activation

Recently, the transplantation of MSCs from different specific tissue sources has been shown to be effective in treating neurodegenerative diseases.^12,36^ Stem cell transplantation strategies based on the secretion of a variety of cytoprotective factors and cell differentiation are essential for treating multiple complex neuropathologies in AD.^37^ We herein demonstrated that hDPSCs have excellent inflammatory regulation and neuroprotective effects in in vitro and in vivo preclinical models of AD. By using an LPS-induced microglia inflammation model and a glutamate-induced HT22 excitotoxicity model, we found that hDPSCs both reduce oxidative stress and apoptosis of damaged cells, thereby contributing to neuroprotection. Furthermore, LY294002 reversed the antioxidant and Nrf2 nuclear accumulation-promoting effects of hDPSCs. Single administration of hDPSCs in the hippocampus of 3xTg-AD mice obviously restored mouse memory and impressively altered neuropathology at multiple critical levels in the AD microenvironment. Further investigation of the molecular mechanisms revealed that the neuroprotective effects of hDPSCs were partially mediated by the suppression of oxidative stress by facilitating nuclear accumulation of Nrf2 via the p-AKT (ser473)/p-GSK3β (ser9) pathway. Taken together, our findings suggested that therapeutic strategies for hDPSCs-mediated AKT/GSK3β/Nrf2 signaling may improve the pathophysiology of AD, promising the treatment of AD (Fig. 8).

As resident macrophages in the brain, microglia are multifunctional processors that maintain central nervous system homeostasis and are critical in early development and aging.^38^ In the pathogenesis and progression of AD, in addition to its own protective clearance role, hyperreactive microglia also produce neurotoxic effects due to excessive production of cytotoxic mediators and pro-inflammatory cytokines, further exacerbating neuronal dysfunction.^39^ Meanwhile, damaged microglia also facilitate persistent neuroinflammation and ROS generation. Given the limited antioxidant capacity, the redox balance of nerve cells is essential for cell survival and microenvironment homeostasis.^40^ Thus, removal of ROS and restoration of microglial homeostasis have been proposed to be central considerations in AD treatment strategies.^41^ The efficacy of hDPSCs on microglia function was investigated in the original study using an LPS-induced BV2 cell model. Co-culture with hDPSCs significantly reduced LPS-mediated microglia pro-inflammatory activation and ROS production while enhancing antioxidant enzymes expression. Importantly, these overall beneficial effects did not simply suppress M1 activation but simultaneously promoted the specific repair process of microglial M2 responses. This neuroprotection is also manifested in neurons in pathological states. As one of the primary hallmarks of AD, it is widely believed that damage or loss of neurons and synapses that underlie cognitive performance has the best correlation with cognitive decline in AD.^42^ Glutamatergic neurons may have been lost early in vulnerable pathways such as the hippocampal networks before classical pathological factors such as Aβ and tau disrupted synapses, leading to oxidative stress induced by excitotoxicity.^26^ Our data showed that in glutamate-induced HT22 cell models, hDPSCs visually scavenged both cellular and mitochondrial ROS and restored mitochondrial structural integrity. Intracellular increased nuclear transduction of the antioxidant transcription factor Nrf2 and reduced oxidative damage, to some extent, reduced apoptosis and stress responses of nerve cells.

It is well known that Nrf2 is a critical mediator of cellular antioxidant defense, and in particular, its abundance in the nucleus determines the activity of relevant antioxidant enzymes downstream under normal and stress conditions.^43^ Consistent with the available research evidence,^9,10^ Nrf2, which is localized predominantly in the cytoplasm in AD models, is distributed evenly between the nucleus and cytoplasm in response to therapeutic induction of hDPSCs, and its expression activity with target genes is significantly enhanced in both mRNA and protein. Thus, it is natural to ask the question of how hDPSCs increase Nrf2 transcriptional activity and nuclear abundance. Our further results indicated that increased expression of Nrf2 is accompanied by the phosphorylation of AKT and deactivation of GSK3β. The PI3K/AKT/GSK3β pathway is an essential signaling transduction pathway involved in diverse biological processes such as cellular growth, survival, and metabolism.^44^ GSK3β is activated under the resting conditions, leading to Nrf2 degradation, while under oxidative stress conditions in AD, the inhibition of GSK3β can induce the activation of Nrf2.^45,46^ Accordingly, we reckoned that the AKT/GSK3β signaling pathway may mediate the promoting effect of hDPSCs on Nrf2 nuclear accumulation and antioxidant proteins expression.

The application of animal models for AD is essential for understanding the pathogenesis and testing the efficacy of preclinical treatment. We therefore used 3xTg-AD mice with typical pathological features, including the deposition of amyloid β-peptide, hyperphosphorylated tau, and increased oxidative stress, as animal model for the study of AD.^47^ The 3xTg-AD mice transplanted with hDPSCs exhibited improved spontaneous exploration and memory performance, as assessed by two well-defined and widely used cognitive (hippocampus-dependent) tasks. The improvement in cognitive function was also reflected in the treatment of pathological features of AD mice by hDPSCs. We found remission of representative pathological manifestations associated with AD in the hippocampus at both the histological and protein levels, including Aβ plaques, hyperphosphorylated Tau protein, neuroinflammation, and oxidative stress. Of note, as another distinctive characteristic of AD, brain atrophy improved markedly following transplantation of hDPSCs, accompanied by decreased expression of the apoptosis marker Bax and cleaved caspase 3. Indeed, the observed polarization of microglia from the M1 phenotype to the anti-inflammatory M2 phenotype in the hippocampus of hDPSC-treated AD mice is consistent with that observed in the cellular model. These outcomes might depend on the p-AKT (ser473)/p-GSK3β (ser9) signaling pathway activated by hDPSCs upregulates the expression of Nrf2, as well as its target genes such as HO-1, thus exerts neuroprotective efficacy in the hippocampus against the progressive AD pathology. However, the study still has some limitations. In vitro cell models do not well reflect the state of neural cells within the brain, which makes it necessary to explore AD treatment strategies with brain organoids that faithfully cover the phenotype of the disease.^48^ Considering the certain paracrine action and multilineage differentiation capacities of MSCs, it is unclear whether there are multi-target therapeutic pathways for hDPSCs to improve the cognitive ability of AD mice. Combining nanotechnology with genetic engineering to track the survival and dynamics of transplanted stem cells in real time while checking the integration of stem cells into host neural circuits in animal models is also emerging as a new strategy to explore AD therapy.^49,50^ Our results reasoned that the underlying mechanism of its repair may be related to promoting oxidative stress homeostasis in the brain. Moreover, we still do not know how hDPSCs stimulate AKT to activate Nrf2 function. This will be investigated in the next studies.

In conclusion, our data supported a therapeutic strategy based on hDPSCs acting as promising neuroprotective mediators, contributing to cognitive and pathological improvement, which may be largely due to hDPSCs exercising their antioxidant effects via AKT/GSK3β-mediated Nrf2 activation and nuclear transduction. Crucially, these findings demonstrated the great potential of hDPSCs in the treatment of AD and provide new horizons and mechanistic basis for determining their clinical applications.

## Materials and methods

### Isolation, culture, and identification of hDPSCs

Third molars with no periodontal disease or dental caries were extracted from healthy volunteers aged between 20 and 30 years from the Department of Oral Surgery, Renmin Hospital of Wuhan University, with ethical approval (Approval Number: WDRY-2022-K025, Wuhan, China). Alcohol (75%) was used to sterilize the freshly collected tooth surfaces. Dental pulp tissue was rinsed three times with PBS and cut into small pieces. After that, the pulp tissue was digested with 4 mg/mL dispase (Sigma, USA) and 3 mg/mL collagenase type I (Gibco, MD) for 20 min at 37 °C. The cellular suspension was collected and cultured in a T-25 culture flask at 37 °C in a 5% CO_2_ humidified atmosphere with Minimum Essential Medium α (α-MEM; Gibco, USA). The culture system was replaced five days later, and then the culture medium was changed every three days. At this time, the passage of cell is passage 0 (P0). After three generations, hDPSCs were seeded in a 10-cm dish marked as P3. Upon 85% confluence, for the detection of cell surface markers, hDPSCs were suspended in flow cytometry staining buffer with FITC-anti-human CD44, 45, 73, and PE-anti-human CD34, 90, and HLA-DR (BD, USA). For trilinear differentiation, hDPSCs were cultured according to the manufacturer’s instructions in the hMSCs-osteogenic, chondrogenic, and adipogenic induced differentiation kit (OriCell, China). After three weeks of induction culture, hDPSCs were severally stained with Alizarin red, HE, Alcian blue, and Oil red O. All isolation, cultivation, identification, quality control, and preservation procedures of hDPSCs were in accordance with quality standards.

### Mice

The 3xTg-AD mice and the corresponding wild type (WT) were fed in a specific pathogen-free (SPF) barrier system including standard pellet diet, pure water, and temperature of 22 ± 2 °C. All procedures performed in studies involving animals were in strict accordance with the US National Institutes of Health Guidelines for the Care and Use of Laboratory Animals and were approved by the Institutional Animal Care and Use Committee (IACUC) of Renmin Hospital of Wuhan University (WDRM20230103D). The mice in each group did not distinguish between sexes.

### Quantitative real-time PCR analysis

Total RNA was isolated from the cells using Trizol Reagent. First-strand cDNA was synthesized by incubating 1 µl of total RNA with oligo dT and reverse transcriptase (Takara, China). The primers are listed in Supplementary Table 1A. All qRT-PCR was performed using the SYBR Green color qPCR supermix (Novoprotein, China) on the Bio-Rad System (Bio-Rad, USA). GAPDH expression was used as normalized internal control. Relative changes in expression levels were calculated using the 2^−(ΔCT,Tg-ΔCT,control)^ method. All analyses were performed in biological triplicates for each sample.

### Western blotting

The cells or mouse hippocampal tissue samples were harvested and lysed in RIPA buffer containing protease inhibitors cocktail. The homogenates were centrifuged at 12 000 × *g* for 20 min at 4 °C. The protein extracts were quantitated by BCA assay, boiled in SDS loading buffer. After SDS-PAGE on 8%–12% Tris gels, the samples were transferred to a PVDF membrane. The membrane was blocked with Tris-buffered saline (TBS) containing 5% nonfat milk and 0.1% Tween 20 (TBST) at room temperature (RT) for 2 h, then blotted with the corresponding primary antibodies overnight at 4 °C, followed by incubation with the secondary antibodies at RT for 1 h, with subsequent detection using ECL substrate. The primary antibodies used in this study are listed in Supplementary Table 1B. Finally, the membrane was imaged with the ImageQuant 800 (GE Healthcare, USA) and quantitatively analyzed with Image J software.

### In vitro co-culture experiment

BV2 and HT22 cells were purchased from Procell and cultured in six-well plates with DMEM supplemented with 10% FBS and 1% penicillin/streptomycin at a density of 2 × 10^5^ cells per well. For control group, BV2 or HT22 cells were cultured with 2 ml complete medium. BV2 or HT22 cells were induced by incubation with LPS (Sigma, China) or L-Glutamic acid (GLU, MCE, China) at a corresponding concentration. Pre-incubate with LY294002 for 2 h prior to LPS or GLU treatment. In the treatment group, a co-culture transwell chamber (0.4 μm pore size; Corning) was used to assess the effects of hDPSCs on LPS-induced BV2 or GLU-induced HT22 cells. hDPSCs were seeded in the upper compartment at a 1 × 10^5^ cells per well in 1 mL of α-MEM complete medium with 24 h.

### Cell viability test

The cytotoxicity of LPS or GLU-induced cells was assessed by cell viability test using CCK-8 assays (Sigma). The three group cells were plated at a concentration of 1 000 cells per well in the 96-well culture plates. The medium that contained 10% FBS was used as blank control. Replications were done with five wells plating the same cells. 10 µL CKK-8 reagent was added to each well of the plate at 0, 12, 24, 48 h after inoculation, and then the plates were incubated for 2 h per day. Last, the absorbance of plates was then measured at 450 nm using a microplate reader (EnSight, PerkinElmer, United States).

### Flow cytometry

For apoptosis analysis, BV2 or HT22 cells were harvested and centrifuged at RT for 5 min. All cells were resuspended with 195 μL of buffer solution and incubated with 5 μL Annexin V-FITC and 10 μL propidium iodide (PI) at RT for 15 min according to the manufacturer’s instructions (Beyotime, China). Finally, the stained cells were analyzed using the FACSCanto II system (BD Biosciences) to determine the ratios of apoptotic cells.

### Detection of mitochondrial superoxide, membrane potential, and ROS generation

ROS detection kit (Beyotime, China) was used to detect and analyze intracellular reactive oxygen species. According to the manufacturer’s instructions, cells exposed to different treatments were collected, and incubated with DCFH-DA at 37 °C for 20 min. Then, the cells were rinsed three times with serum-free medium. Finally, DCF fluorescence of cells was detected by a fluorescence microscope at an excitation wavelength of 488 nm and an emission wavelength of 525 nm.

Mitochondrial superoxide levels were determined using MitoSox Red Indicator (Yeasen, China). 5 mM of MitoSOX reagent was diluted in PBS to 5 μmol/L of mitoSOX reagent working solution. Then, 1.0 mL 5 μM MitoSOX reagent working solution was applied to cover cells adhering to coverslips at 37 °C for 10 min. The cells were gently washed three times with warm buffer. Specimens were examined under a fluorescence microscope.

Induced HT22 were seeded in six-well plates. The mitochondrial membrane potential assay kit with JC-1 (Beyotime, China) was used to detect the mitochondrial membrane potential. The ratio of red/green fluorescence intensity (aggregates/monomers) representing the extent of mitochondrial membrane potential depolarization was calculated using the ImageJ software.

### Immunofluorescence

The induced cells were seeded onto coverslips, and the mouse brain tissues were fixed in 4% formaldehyde for 30 min or 3 days. Then incubated with 0.5% Triton X-100 for 10 min and blocked with 2% BSA for 0.5 h at RT. All samples were then incubated with primary antibodies used in this study are listed in Supplementary Table 1B. After overnight incubation at 4 °C, the samples were incubated with Alexa Fluor® 488-, or 594-conjugated secondary antibodies for 1 h at 37 °C, counterstained with DAPI, then images were taken with a fluorescence microscope and quantitatively analyzed with ImageJ software.

### Electron microscopy

Cell or mouse tissue samples were collected, fixed in 2.5% glutaraldehyde at RT for 2 h, and then stored at 4 °C. The specimens were rinsed in 0.1% glutaraldehyde buffer for 2 h and followed by post-fixing with 1% OsO_4_ at 4 °C for 1 h. The specimens were secondly dehydrated with graded acetone and finally embedded in epoxy resin. Ultrathin sections were observed under an HT7800 electron microscope (Hitachi, Japan).

### Intracerebroventricular injections of hDPSCs

10-month-old mice were anesthetized using 1% pentobarbital sodium (0.1 mL per 20 g). The intraventricular injections were performed using a stereotaxic apparatus (RWD, China) at the following coordinates: anteroposterior (AP) −2.06 mm, mediolateral (ML) ± 1.5 mm, dorsoventral (DV) −1.8 mm from bregma. PBS (5 μL) or hDPSCs (5 μL, 1 × 10^5^ cells) was bilaterally injected into the hippocampus at a speed of 80 nL per min. After the injection, the needle was placed in situ for an additional 5 min and then slowly removed (3 min). The mice were placed on a heating pad until they were awake and given postoperative care.

### Morris water maze test (MWM)

All mice were tested 1 month after hDPSCs or PBS transplantation. The mice were placed in the test chamber for 2 h in advance. The MWM (1.2 m in diameter) filled with milky water maintained at 22 °C, with an escape platform anchored 1 cm below the water surface in the NW quadrant. During the training (days 1 to 6), the mice were placed in the water in a random quadrant (SW, SE, or NE) and given 60 s to locate the hidden platform, with an interval of 15-min. If mice failed to arrive at the platform within 60 s, they will be manually guided there. On day 7, a probe trial with no platform was presented, with a percentage of time spent in the quadrant that previously contained the escape platform during task acquisition exceeding 60 s. The latency in all trials were analyzed by means of video.

### Fear conditioning test (FCT)

Animals were tested in the fear conditioning device and 30 s afterward subjected to 3 pairs of contexts (conditioned stimulus—20 s, 2 kHz, 75 dB; 20 s, 2 000 lux) that co-terminated with an aversive unconditional stimulus (electric stimulus—2 s, 0.4 mA). The training period was 60 s and was repeated twice. In the second day, mice were placed for context without any shocks to test long-term memory. Freezing was scored automatically by the machine (Superfcs, China) and the freezing detection threshold was the same for all mice during the test.

### Statistical analysis

Data are represented as mean ± SD. GraphPad Prism (version 9.0) is used to analyze three or more independent experiments. Statistical analyses were performed using either Student’s t-test (two-group comparison) or one-way ANOVA followed by Tukey’s or least significant difference (LSD) for post hoc test (more than two groups), with *P* values less than 0.05 were considered a significant difference.

### Supplementary information

Supplementary information

## Data Availability

All data needed to evaluate the conclusions in the paper are present in the paper and/or the Supplementary Materials.
