# Supplementary material for: Human dental pulp stem cells mitigate the neuropathology and cognitive decline via AKT-GSK3β-Nrf2 pathways in Alzheimer’s disease
Source: Int J Oral Sci. 2024 May 13;16:40. doi: 10.1038/s41368-024-00300-4 (PMC11091120; doi:10.1038/s41368-024-00300-4)
Supplement: Supplementary file 1 — Supplementary information [file 41368_2024_300_MOESM1_ESM.docx]

**Supplementary information**

**Human dental pulp stem cells mitigate the neuropathology and cognitive decline via the AKT-GSK3****β-Nrf2 pathways in Alzheimer's disease**

**Wei Xiong^1, †^, Ye Liu^1, †^,** **Heng Zhou^1^, Junyi Li^1^, Shuili Jing^1^, Cailei Jiang^3^, Mei Li^2^, Yan He^3, *^, Qingsong Ye^1,4, *^**

**Supplementary Figures**

**
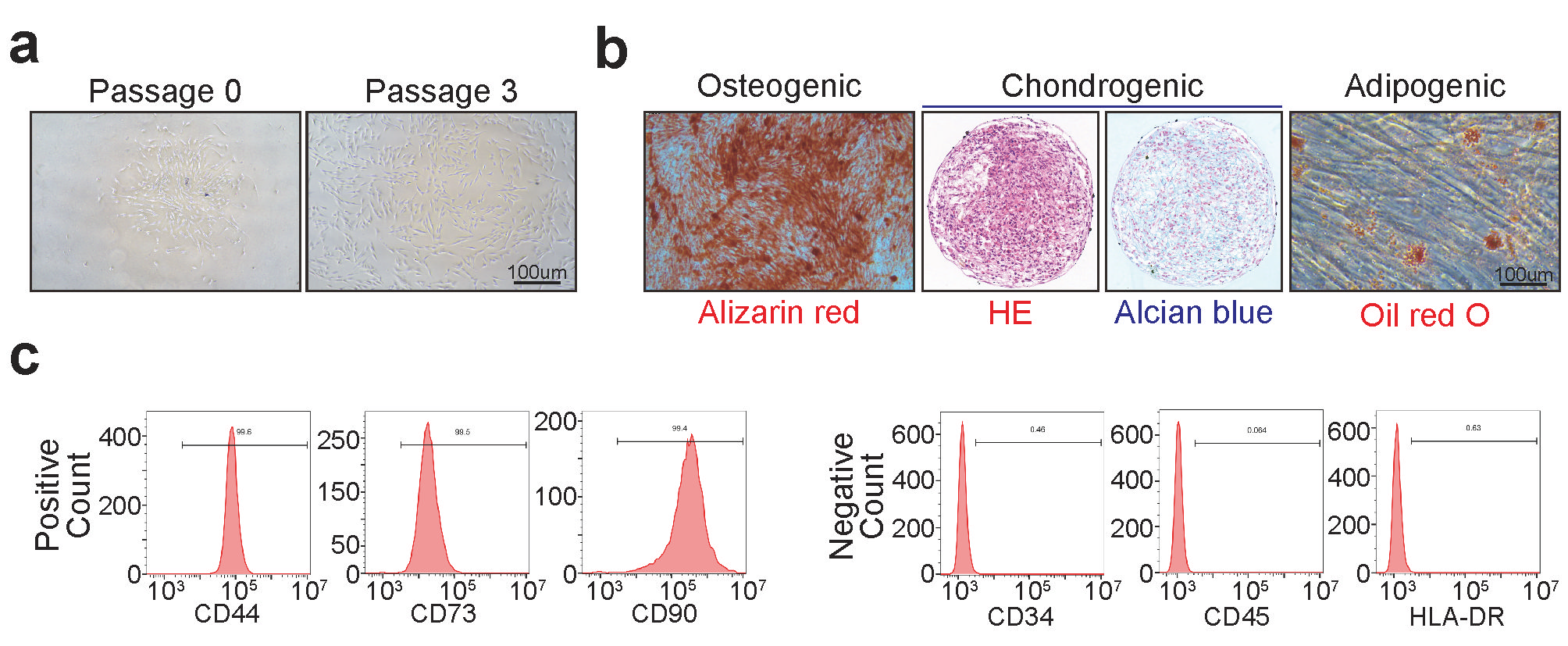
**

**Figure S1.** **The morphology, differentiation potential, and phenotype of clinical-grade hDPSCs were characterized**. (a) The morphology of hDPSCs (P0 or P3) were showed under light microscopy. Scale bar=100 um. (b) The multi-lineage differentiation potential of hDPSCs were confirmed. (c) Flow cytometry revealed hDPSCs were positive for mesenchymal lineage markers (CD44, CD73 and CD90), negative for hematopoietic markers (CD34, CD45 and HLA-DR).


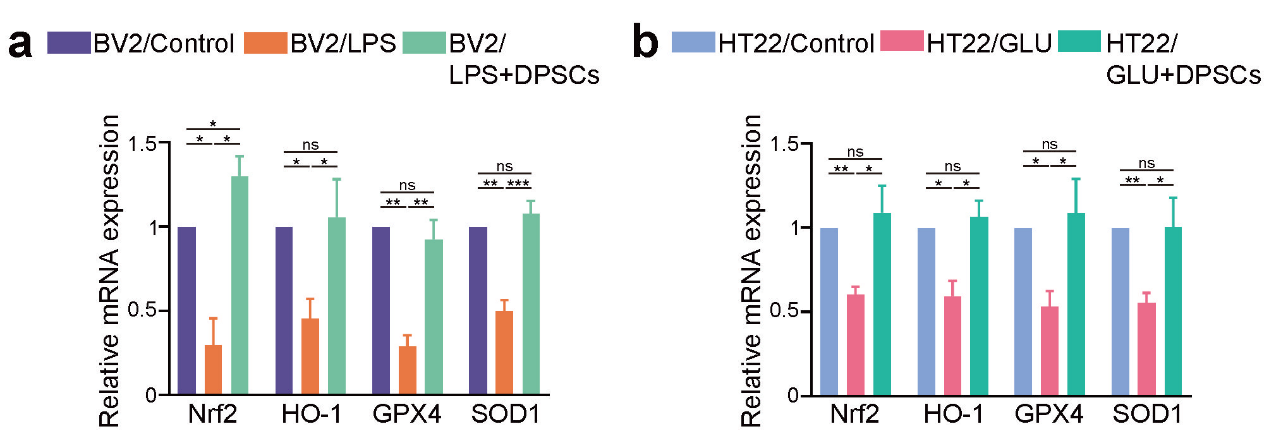


**Figure S2.** **hDPSCs promoted mRNA expression of antioxidation-related genes in AD cell models.** (a, b) Quantitative real-time PCR analysis was performed for the relative mRNA expression of Nrf2, HO-1, GPX4, and SOD1 in the BV2 and HT22 cells models, respectively. (n=3 per group; Values represented mean ± SD; ns indicates no significant, *P < 0.05, **P < 0.01, ***P < 0.001).


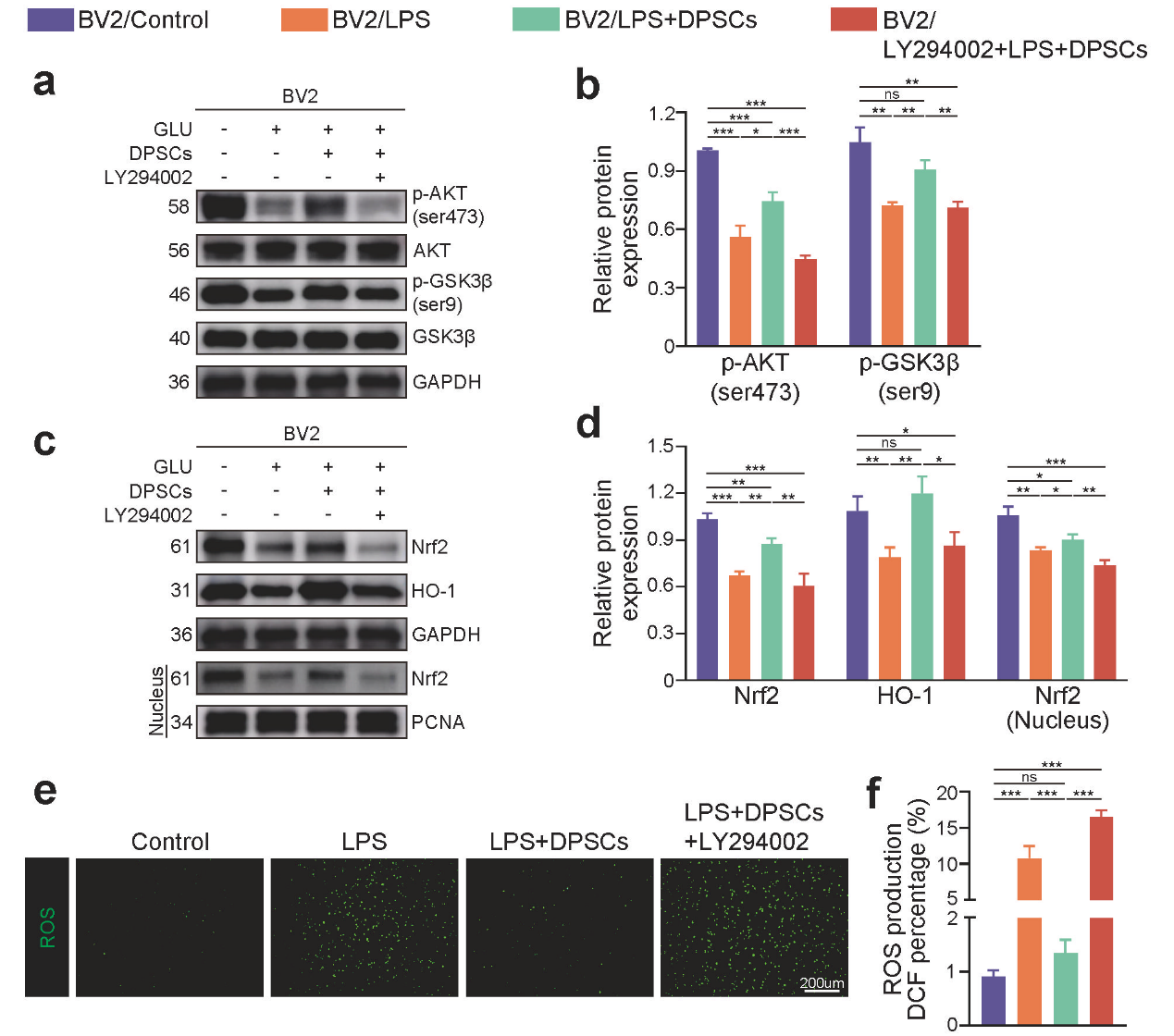


**Figure S3. hDPSCs ameliorated LPS-induced oxidative stress and apoptosis in BV2 cells by activating Nrf2 via the AKT/GSK3β pathway.** (a, b) Representative images and quantification of western blotting showed the expression of total Nrf2, HO-1, and nuclear Nrf2 in different treated BV2 cells. (c, d) Representative western blotting results showed the expression of p-AKT (ser473) and p-GSK3β (ser9) of BV2 cells in Control, LPS, LPS+hDPSCs, and LY294002+LPS+hDPSCs groups. (d) The quantification of p‐AKT (ser473) and p‐GSK3β (ser9) respectively compared with total‐AKT and total‐GSK3β. (e, f) The reactive oxygen species (ROS, green) level in BV2 cells detected by DCFH-DA staining and statistically analyzed. Scale =200 um. (n=3 per group; Values represented mean ± SD; ns indicates no significant, *P < 0.05, **P < 0.01, ***P < 0.001).


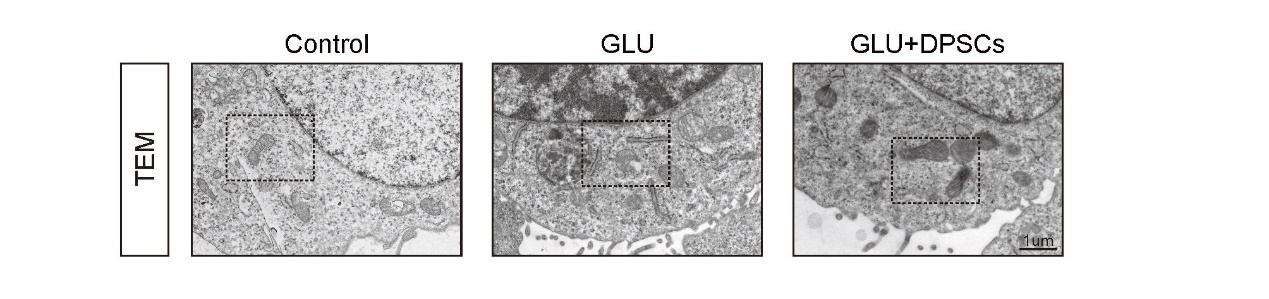


**Figure S4. hDPSCs ameliorated mitochondria damage in GLU-induced HT22 cells.** Morphometric ultrastructural analyses by TEM showed the intracellular mitochondrial structure of HT22 in the three groups. The black box represents the area in Figure 3K. Scale bar =1 um.


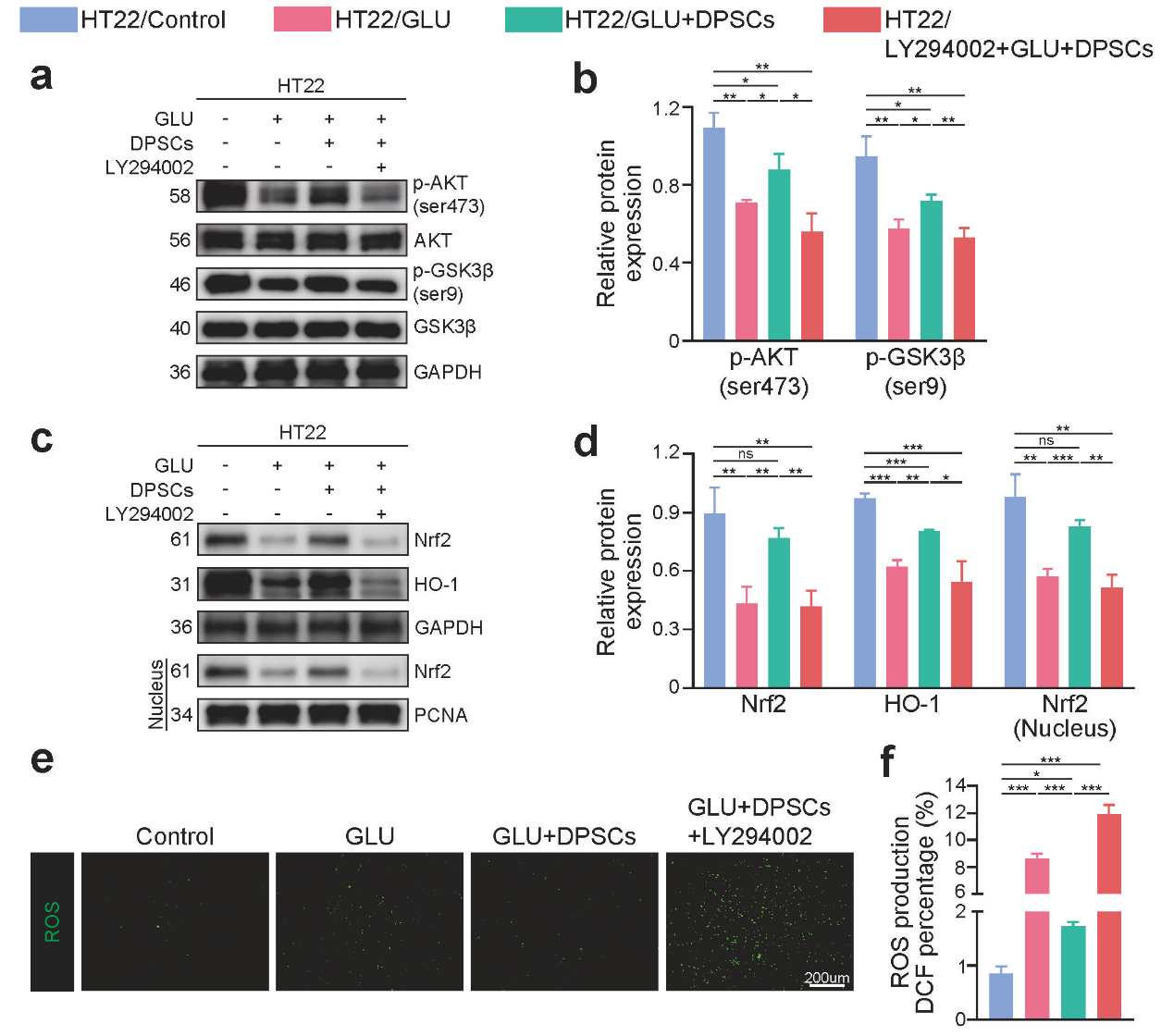


**Figure S5. hDPSCs attenuated apoptosis in GLU-induced HT22 cells by activating Nrf2 via the AKT/GSK3β pathway.** (a, b) Representative images and quantification of western blotting showing the expression of total Nrf2, HO-1, and nuclear Nrf2 in different treated HT22 cells. (c) Representative western blotting results showed the expression of p-AKT (ser473) and p-GSK3β (ser9) of HT22 cells in the four groups. (d) The quantification of p‐AKT (ser473) and p‐GSK3β (ser9) respectively compared with total‐AKT and total‐GSK3β. (e, f) The reactive oxygen species (ROS, green) level in HT22 cells detected by DCFH-DA staining and statistically analyzed. Scale =200 um. (n=3 per group; Values represented mean ± SD; ns indicates no significant, *P < 0.05, **P < 0.01, ***P < 0.001).


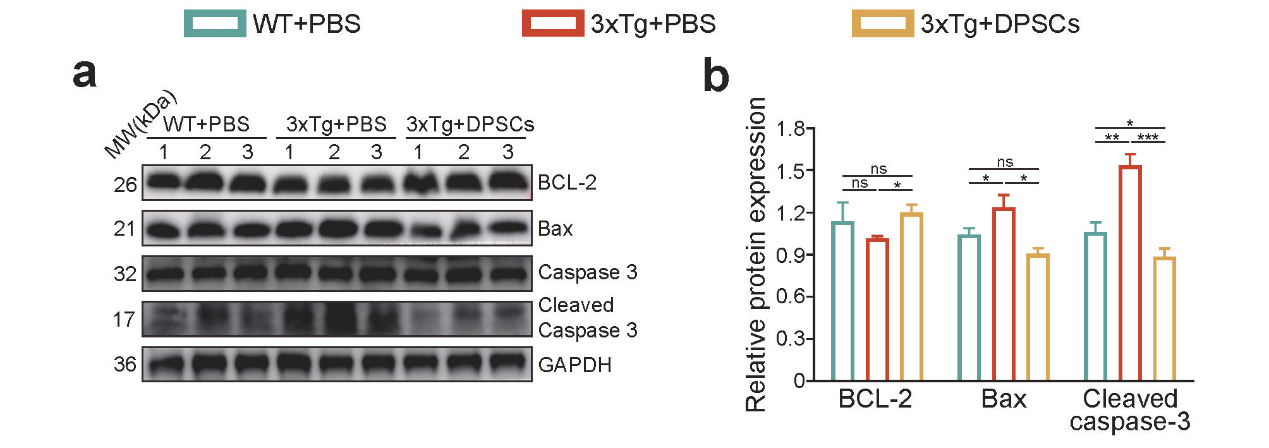


**Figure S6. hDPSCs reduced the apoptosis of hippocampal cells in 3xTg-AD mice.** (a, b) The protein expression and quantification of apoptosis-associated proteins BCL-2, Bax, Caspase 3, and cleaved caspase-3 in the hippocampus. (n=3 per group; Values represented mean ± SD; ns indicates no significant, *P < 0.05, **P < 0.01, ***P < 0.001).


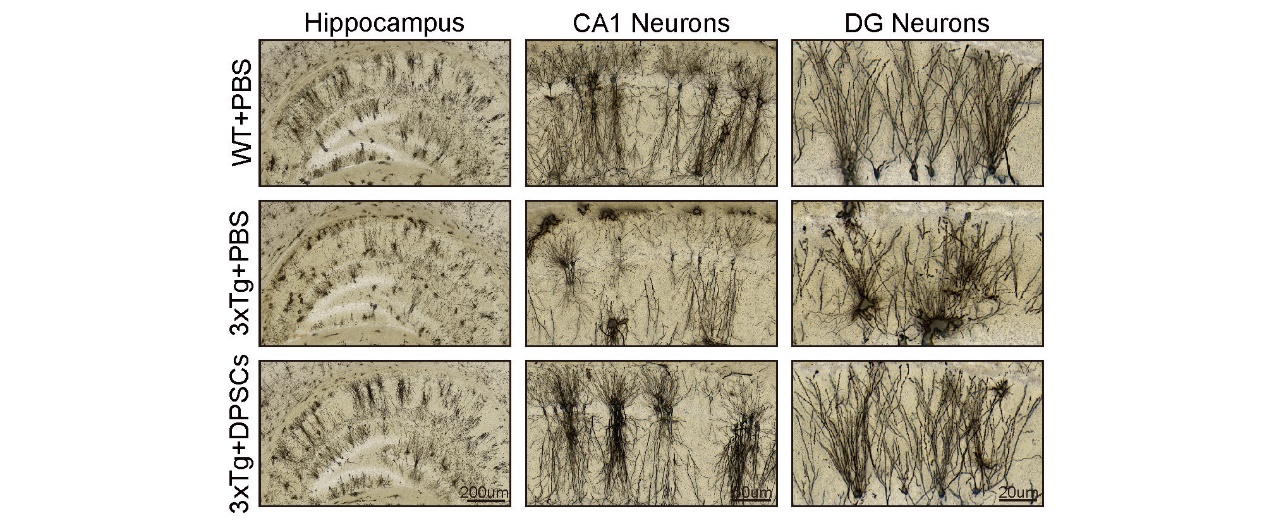


**Figure S7. hDPSCs reduced neuropathology in the hippocampus of 3xTg-AD mice.** Representative Golgi staining of dendritic spine in the CA1 and DG of the hippocampus. Scale bar=200um, 50um, 20um.


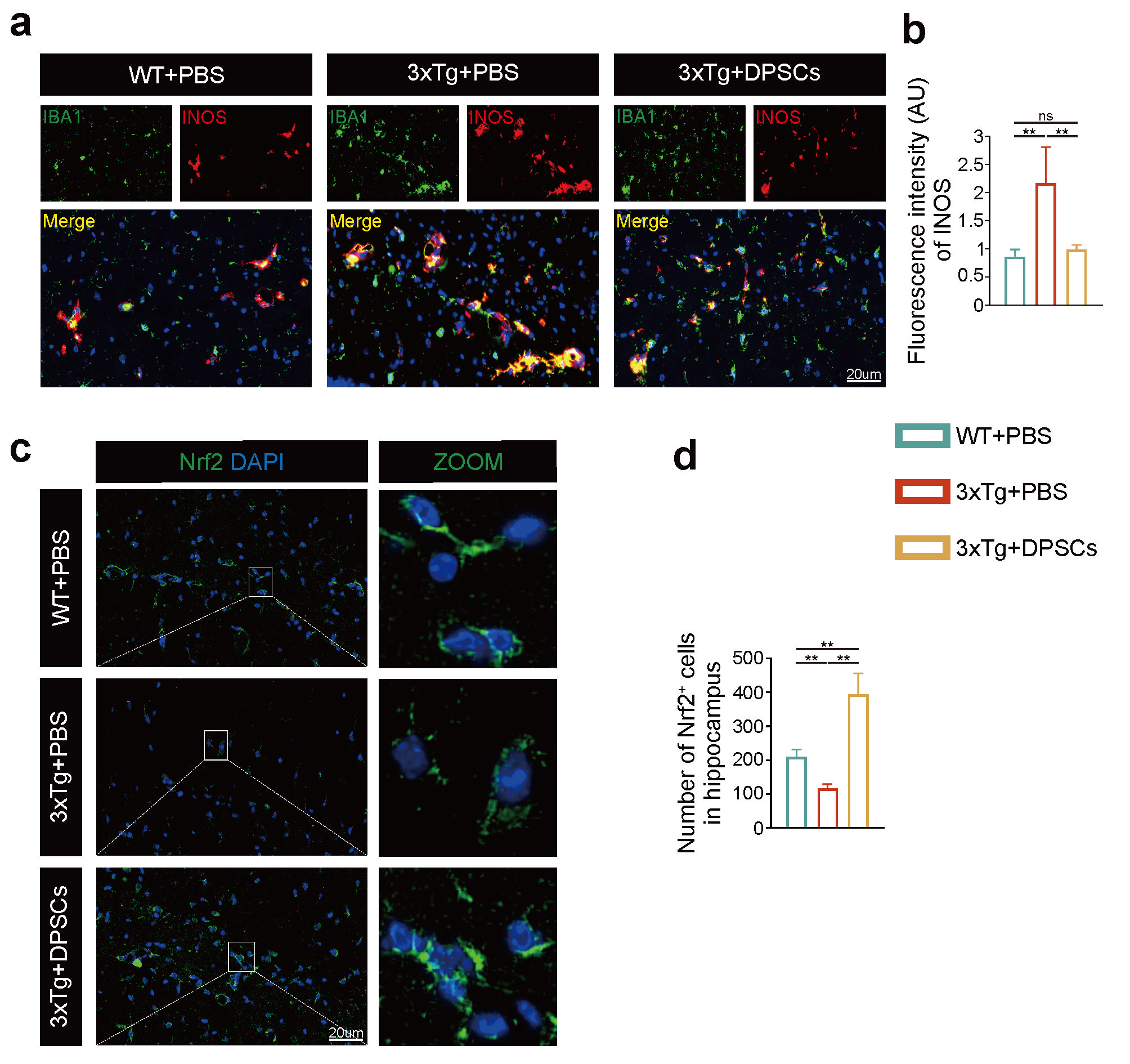


**Figure S8. Neuroprotective efficacy of hDPSCs in 3xTg-AD mice were associated with enhancing Nrf2 nuclear accumulation via AKT/GSK3β pathway.** (a, b) Immunofluorescence staining images and quantification of microglia (IBA1, INOS) and IBA1-INOS co-localization (Merge, M1 microglia) in the hippocampus between three groups mice. Scale =20 um. (c, d) Representative immunofluorescent staining images and quantification of Nrf2 in the hippocampus between three groups mice. Scale =20 um. (n=3 per group; Values represented mean ± SD; ns indicates no significant, **P < 0.01).

**Supplementary Table 1A. Primers and interference sequences used in this study**

| Gene | Forward primer | Reverse primer | Purpose |
| --- | --- | --- | --- |
| TNF-α  IL-6  IL-1β  IL-10  Nrf2  HO-1  GPX4  SOD1  GAPDH | 5’-GACGTGGAACTGGCAGAAGAG-3’  5’-TAGTCCTTCCTACCCCAATTTCC-3’  5’- GCAACTGTTCCTGAACTCAACT-3’  5’-GCTCTTACTGACTGGCATGAG-3’  5’- GCCACCGCCAGGACTACAG-3’  5’- AGACCGCCTTCCTGCTCAAC-3’  5’- CCCGATATGCTGAGTGTGGTTTAC-3’  5’- TCGGCTTCTCGTCTTGCTCTC-3’  5’- GGCAAATTCAACGGCACAGTCAAG-3’ | 5’-TTGGTGGTTTGTGAGTGTGAG-3’  5’-TTGGTCCTTAGCCACTCCTTC-3’  5’- ATCTTTTGGGGTCCGTCAACT-3’  5’-CGCAGCTCTAGGAGCATGTG-3’  5’- AACTTGTACCGCCTCGTCTGG-3’  5’- GACGAAGTGACGCCATCTGTG-3’  5’- TTTCTTGATTACTTCCTGGCTCCTG-3’  5’- TCGGCTTCTCGTCTTGCTCTC-3’  5’- TCGCTCCTGGAAGATGGTGATGG-3’ | RT-PCR  RT-PCR  RT-PCR  RT-PCR  RT-PCR  RT-PCR  RT-PCR  RT-PCR  RT-PCR |

**Supplementary Table 1B. Antibodies used in this study**

| **Antibody** | **Cat No:** | **Company** | **Species** | **Application** |
| --- | --- | --- | --- | --- |
| TNF-α | 60291-1-Ig | Proteintech | Mouse | WB |
| IL-6 | 21865-1-AP | Proteintech | Rabbit | WB |
| IL-1β | GB111113 | Servicebio | Rabbit | WB |
| IL-10 | 60269-1-Ig | Proteintech | Mouse | WB |
| Arg1 | 16001-1-AP | Proteintech | Rabbit | IF |
| INOS | 18985-1-AP | Proteintech | Rabbit | IF |
| Nrf2 | 16396-1-AP | Proteintech | Rabbit | WB/IF |
| HO-1 | 66743-1-Ig | Proteintech | Mouse | WB |
| GPX4 | 67763-1-Ig | Proteintech | Mouse | WB |
| SOD1 | sc-101523 | Santa crus | Mouse | WB |
| BCL-2 | #3498S | CST | Rabbit | WB |
| Bax | 50599-2-Ig | Proteintech | Rabbit | WB |
| Caspase 3 | 19677-1-AP | Proteintech | Rabbit | WB |
| Cleaved caspase 3 | #9661S | CST | Rabbit | WB |
| AKT1/3 | 60203-1-Ig | Proteintech | Mouse | WB |
| p-AKT (Ser473) | 28731-1-AP | Proteintech | Rabbit | WB |
| GSK3β | ab32391 | Abcam | Rabbit | WB |
| p-GSK3β (Ser9) | #9323S | CST | Rabbit | WB |
| APP | 25524-1-AP | Proteintech | Rabbit | WB |
| p-Tau (AT8) | MN1020 | Invitrogen | Mouse | WB |
| Tau-5 | ab80579 | Abcam | Mouse | WB |
| Aβ | ab201060 | Abcam | Rabbit | IF |
| IBA1 | ab178846 | Abcam | Rabbit | IF |
| GAPDH | 60004-1-Ig | Proteintech | Mouse | WB |
| PCNA | 10205-2-AP | Proteintech | Rabbit | WB |
